# The current status, trends, and challenges of Alzheimer’s disease and other dementias in Asia (1990–2036)

**DOI:** 10.3389/fpubh.2025.1583339

**Published:** 2025-06-10

**Authors:** Ziyang He, Haoqin Zhang, Guishan Hu, Yinan Qiao, Can Yin, Jianqiao Li, Hong Lin, Anguo Wu, Dalian Qin, Betty Yuen-Kwan Law, Guangqiang Hu, Lu Yu

**Affiliations:** ^1^Sichuan Key Medical Laboratory of New Drug Discovery and Druggability Evaluation, School of Pharmacy, Southwest Medical University, Luzhou, China; ^2^Clinic Medical College, Southwest Medical University, Luzhou, China; ^3^Department of Medical Imaging, Southwest Medical University, Luzhou, China; ^4^Department of Anatomy, School of Basic Medical Sciences, Southwest Medical University, Luzhou, China; ^5^State Key Laboratory of Quality Research in Chinese Medicine, Macau University of Science and Technology, Taipa, Macao SAR, China

**Keywords:** Alzheimer’s disease and other dementia, global burden of disease, risk factors, prevalence, mortality, disability-adjusted life years

## Abstract

**Background:**

With global aging, Alzheimer’s disease (AD) and other dementias have emerged as significant health threats to the older adults, garnering considerable attention due to their impact on public health. Despite the substantial burden of dementia in Asia, targeted research remains limited. This study aims to assess the current burden, future trends, risk factors, and inequalities in Asia.

**Method:**

The GBD 2021 study was utilized to evaluate the numbers and age-standardized rates (ASRs) of prevalence, mortality, and disability-adjusted life-years (DALYs) of AD and other dementias from 1990 to 2021. Joinpoint regression analysis was performed to assess the trends during this period, while the Autoregressive Integrated Moving Average (ARIMA) model was employed to predict future trends. Additionally, the relationship between disease burden and sociodemographic index (SDI) was also analyzed.

**Results:**

In 2021, Asia experienced a 250.44% increase in prevalent cases, a 297.34% rise in mortality, and a 249.54% surge in DALYs for AD and other dementias compared to 1990. Meanwhile, the age-standardized prevalence rate, age-standardized mortality rate, and age-standardized DALY rate also exhibited varying degrees of rise from 1990 to 2021. Demographically, the disease burden was higher in women and those aged 65 and above. Regionally, the burden was highest in East Asia and relatively low in South and Central Asia. Nationally, China, India, Japan, and Indonesia reported the most cases. Over the next 15 years, the age-standardized prevalence rate in Asia is expected to peak in 2028 before declining, while the age-standardized mortality rate is anticipated to keep rising. An overall “V” shaped association was found between sociodemographic index (SDI) and the age-standardized DALY rate in Asia. Only smoking, high fasting plasma glucose (FPG), and high BMI were identified as causal risk factors within the GBD framework.

**Conclusion:**

The burden of AD and other dementias in Asia has significantly increased over the past three decades and is expected to persistently impact Asian populations, particularly in developing countries experiencing rapid demographic shifts. Women and the older adult should be a focus of attention. It is imperative to implement targeted prevention and intervention strategies, enhance chronic disease management, and control risk factors.

## 1 Introduction

Alzheimer’s disease (AD) is the most common form of dementia (accounting for approximately 60–80% of cases), with other major forms including vascular dementia (VaD), dementia with Lewy bodies (DLB), frontotemporal dementia (FTD), and mixed dementia (1). As the major cause of senile dementia in the world, AD is a chronic and progressive neurodegenerative disorder that gradually impairs a person’s cognitive abilities, ultimately leading to senile dementia. The main clinical manifestations of senile dementia include a decline in memory, as well as difficulties in thinking, language, and problem-solving abilities (2). The pathological and physiological mechanisms of AD are complex, and its pathogenesis is still unclear. Microscopically, the excessive accumulation of neurotic plaques formed by amyloid-beta (Aβ) peptide and neurofibrillary tangles (NFTs) composed of hyperphosphorylated tau protein are the main pathological features of AD. Moreover, neuronal damage and destruction are also critical neuropathological alterations in the brain of AD (2, 3).

Dementia has emerged as an increasingly severe global public health issue, imposing a heavy economic and disease burden on societies and families. According to the data from the World Health Organization (WHO), in 2019, over 55 million people had dementia globally, posing an estimated economic burden of 1.3 trillion dollars (3). Furthermore, recent data indicates that the number of people with dementia is anticipated to be 152.8 million in 2050 (4). The Global Burden of Diseases, Injuries, and Risk Factors (GBD) Study 2021 employed a systematic approach to analyze and quantify the epidemiological data of 371 diseases across 204 countries and territories, as well as subnational levels, including AD and other dementia. Relevant data from the GBD database indicate that among disorders affecting the nervous system, AD and other dementias are significant contributors to the global burden of disability-adjusted life-years (DALYs). In 2021, AD and other dementias ranked 25th globally in the list of disease causes in terms of DALYs, accounting for 36.3 (17.2–77.4) million DALYs, a substantial increase of 168.7% compared to the figures from 1990 (5, 6). In addition, AD and other dementias ranked 8th in age-standardized mortality rate (ASMR), estimated at 25.2 (6.36–65.6) per 100,000 population, causing a global death toll of 1.96 (0.50–5.12) million individuals (7).

Previous research on the burden of dementia has predominantly focused on a global or national scale, with a dearth of comprehensive regional analyses, particularly in Asia. This study aims to utilize data from the GBD 2021 to systematically analyze the disease burden of AD and other dementias in Asia and to assess the risks of dementia across different countries and territories. By doing so, it seeks to provide crucial scientific evidence for developing integrated disease prevention and control strategies, proactively address the challenges posed by an aging society, reveal health inequities between different countries and regions, and foster international cooperation and exchange.

## 2 Materials and methods

### 2.1 Case definition and data sources

The data for this study were derived from the GBD Study 2021 dataset, which can be obtained from the Institute for Health Metrics and Evaluation (IHME). The GBD is a comprehensive and multinational research project that covers diseases and injuries worldwide, providing detailed data including incidence, prevalence, mortality, and DALYs, which together constitute a comprehensive assessment of the disease burden. To ensure data quality, all data used by the GBD institute undergo thorough screening. The study employs the latest epidemiological data and enhanced standardization methods through collaboration with research institutions and experts from various countries, ensuring the breadth and accuracy of the data. The GBD study 2021 conducted an in-depth assessment of 371 diseases, injuries, and functional impairments, as well as 88 risk factors, covering 204 countries and territories, and 811 subnational locations, providing annual data from 1990 to 2021. In this study, the certainty and 95% uncertainty intervals for the prevalence, mortality, and DALYs of AD and other dementias were obtained from the GBD 2021 data.^1^ In the GBD, the reference case definition for each disease is either the most commonly used or derived from the latest consensus. Data based on alternative case definitions were adjusted if systematic bias was detected. For the analysis concerning AD and other dementias, the reference definition of the disease is from the International Classification of Diseases (ICD, ICD-9, ICD-10).

In this study, when we analyzed specific countries and regions in Asia, we found that the GBD 2021 database included a total of five GBD regions: Central Asia, High-Income Asia Pacific, South Asia, East Asia, and Southeast Asia. Among them, the Central Asia region consists of 9 countries, the High-Income Asia Pacific region has 4 countries, the South Asia region includes 5 countries, the East Asia region comprises 2 countries and 1 region, and the Southeast Asia region has 13 countries.

Ethical approval was exempt as the data were fully anonymized and publicly accessible.

### 2.2 Estimation framework

The detailed description of the methods for estimating the prevalence, mortality, and DALYs of AD and other dementias can be found in advanced journals or research reports (6–8). This study adheres to the methodological framework of the GBD study and conducts a comprehensive analysis of the disease burden of AD and other dementias in the Asian region. Differences in gender and age ranges are also assessed. We employed an analysis method that covers the entire age spectrum, which can be found in relevant literature (9, 10). When we downloaded the data, we selected 20 age groups for population stratification, including “<5 years, 5–9 years, 10–14 years, 15–19 years, 20–24 years, 25–29 years, 30–34 years, 35–39 years, 40–44 years, 45–49 years, 50–54 years, 55–59 years, 60–64 years, 65–69 years, 70–74 years, 75–79 years, 80–84 years, 85–89 years, 90–94 years, 95 + years.” All subsequent analyses were based on the criteria described above.

All available mortality data codes have been calibrated to ensure accuracy and are used to estimate death rates related to AD and other dementias. The Cause of Death Ensemble model (CODEm) is a modeling tool specifically developed for GBD research and is used in conjunction with alternative strategies to accurately estimate these death rates, particularly in situations with insufficient data, substantial changes in reporting, or unusual epidemiology. DisMod-MR 2.1 is a Bayesian meta-regression tool used in GBD to estimate disease prevalence for non-fatal health outcomes. DALYs is a measure of the overall burden of disease, injury, or risk factors, which combines Years Lived with Disability (YLD) and Years of Life Lost (YLL) to quantify the health loss caused by specific diseases and injuries. To ensure comparability of data across different regions and time periods, we use age-standardized rates based on the world standard population. The disease burden estimates reported in GBD studies are accompanied by a 95% uncertainty interval (UI), which reflects the probability that the true parameter value falls within this interval. The UI takes into account not only the variability of parameter estimates but also the uncertainty arising from data collection, model selection, and other estimation processes. The Socio-demographic index (SDI) is a composite measure used to assess the level of development in a country or region, constructed from three indicators: lag-distributed income per capita, average years of education, and total fertility rate among women below 25 years old. SDI ranges from 0 to 1, with higher values indicating a better development status of a country or region. Based on the 2021 SDI values, countries worldwide are divided into five SDI regions: low, low-middle, middle, high-middle, and high SDI countries. Through SDI, researchers can compare the health status of different countries and regions at different levels of development and track changes in health outcomes over time. Risk factors are assessed at four levels. The risk factors we analyzed include smoking, high fasting plasma glucose (FPG), and high body-mass index (BMI). The percentage of deaths and DALYs related to this disease can be found in the GBD results tool. This study adopted the previously established definitions for these risk factors.

### 2.3 Statistical analysis

In this study, R software (version 4.3.3) and GraphPad Prism 8 software were used for analysis and visualization, specifying “Asia” as the geographical location and selecting “Alzheimer’s disease and other dementias” as the cause of the study. We presented the changes in age-standardized prevalence rate, age-standardized mortality rate, age-standardized DALY rate, number of cases, mortality, and DALYs from 1990 to 2021 in the form of tables and figures to illustrate the trends in the burden of dementia. Additionally, this study also provided the distribution of these indicators at the national, regional, gender, and age levels in Asia. All hypothesis tests were two-tailed, and a *p*-value less than 0.05 was considered statistically significant.

We utilized Joinpoint regression software (version 5.1.1.0) to analyze the time trends of AD and other dementias in Asia from 1990 to 2021. The principle of this statistical method is to utilize the least squares approach to estimate the patterns of change in the relevant indicators, thereby circumventing the subjectivity often associated with typical trend analyses based on linear trends, aiding in identifying significant joinpoints where there are notable changes in the trend. The approach involves segmenting the trend into multiple phases and calculating the annual percent change (APC) and its 95% confidence interval (CI) for each phase. The average annual percent change (AAPC) is then used to summarize the overall trend from 1990 to 2021. An APC greater than 0 indicates an increasing trend, while a negative APC indicates a decreasing trend. To assess whether the trends are statistically significant, we examined the *p*-values. A *p*-value below 0.05 suggests that the trend is significant, whereas a *p*-value above 0.05 indicates that the trend is stable.

The Autoregressive Integrated Moving Average (ARIMA) model, a prevalent analytical technique for time series data, was employed in this investigation to forecast the disease burden of AD and other dementia from the years 2022 to 2036. Comprehensive methodological insights can be gleaned from prior scholarly works (11, 12). The ARIMA model consists of an autoregressive (AR) model and a moving average (MA) model. The basic assumption is that the data sequence is a time-dependent stochastic variable, and its autocorrelation can be described by the ARIMA model, which can be used to predict future values based on past values. To elaborate on the methodology: The ARIMA model amalgamates autoregressive elements, which capture the serial dependence through lagged values, with moving average components that account for the error terms’ autocorrelations. The model’s configuration is articulated as ARIMA (p, d, q), where “p” is the count of autoregressive terms, “d” is the level of differencing applied to achieve stationarity, and “q” is the order of moving average terms that models the impact of past forecast errors on the current one. This structured approach allows for a nuanced understanding and forecasting of time series data. The stationarity of the data was assessed using the Augmented Dickey-Fuller (ADF) test, while the Akaike Information Criterion (AIC) and Bayesian Information Criterion (BIC) were employed to select the optimal ARIMA model for predicting the disease burden from 2022 to 2036. The Ljung-Box test was utilized to examine whether the model residuals exhibited autocorrelation during the diagnostic phase of the ARIMA model (13). In terms of method selection, while more advanced models could certainly be considered, we opted for the ARIMA model due to its transparency and computational efficiency. Given our primary focus on establishing a robust baseline forecast, we felt that the ARIMA model was the most appropriate choice for our purposes.

## 3 Results

### 3.1 Temporal distribution of AD and other dementias in Asia

#### 3.1.1 Overview

As depicted in Table 1, in the Asian region, the age-standardized prevalence rate of AD and other dementias increased from 624.53 (95% UI: 544.79–713.56) per 100,000 population in 1990 to 706.22 (95% UI: 610.79–812.27) per 100,000 population in 2021. In terms of prevalence cases, there was a rise from 9,090,622 (95% UI: 7,888,142–10,340,205) in 1990 to 31,857,009 (95% UI: 27,609,350–36,564,142) in 2021. The number of deaths attributable to AD and other dementias in Asia increased from 258,452 (95% UI: 62,058–683,801) in 1990 to 1,026,939 (95% UI: 267,220–2,616,593) in 2021. Concurrently, the age-standardized mortality rate of AD and other dementias in Asia grew from 24 per 100,000 population (95% UI: 5.89–64.22) to 25.66 per 100,000 population (95% UI: 6.84–65.67). The DALYs attributed to AD and other dementias in the Asian region escalated from 5,726,844 (95% UI: 2,671,161–12,706,695) in 1990 to 20,017,626 (95% UI: 9,585,679–42,792,471) in 2021. Correspondingly, the age-standardized DALY rate of AD and other dementias in Asia increased from 428.61 per 100,000 population (95% UI: 194.99–935.15) to 460.48 per 100,000 population (95% UI: 218.29–981.01). Additionally, from 1990 to 2021, the data for AD and other dementias indicated a higher burden in women compared to men.

**Table 1:** Prevalence, mortality, and DALYs of Alzheimer’s disease and other dementias in Asia.

Year	Male	Female	Both
1990			
Prevalence (95% UI)	3,518,228 (3,015,436, 4,019,266)	5,572,394 (4,845,238, 6,355,122)	9,090,622 (7,888,142, 10,340,205)
Mortality (95% UI)	87,915 (20,450, 244,845)	170,537 (41,429, 448,399)	258,452 (62,058, 683,801)
DALYs (95% UI)	2,093,325 (965,365, 4,756,647)	3,633,519 (1,708,521, 7,928,193)	5,726,844 (2,671,161, 12,706,695)
ASPR (95% UI) per 100,000	520.96 (452.87, 597.76)	701.57 (611.83, 799.54)	624.53 (544.79, 713.56)
ASMR (95% UI) per 100,000	18.58 (4.51, 51.84)	27.5 (6.74, 72.23)	24 (5.89, 64.22)
ASDR (95% UI) per 100,000	339.75 (152.6, 760.44)	491.64 (225.91, 1060.08)	428.61 (194.99, 935.15)
2021			
Prevalence (95% UI)	11,844,942 (10,076,621, 13,577,622)	20,012,067 (17,369,976, 22,900,589)	31,857,009 (27,609,350, 36,564,142)
Mortality (95% UI)	336,042 (81,691, 912,169)	690,897 (184,954, 1,751,094)	1,026,939 (267,220, 2,616,593)
DALYs (95% UI)	7,061,588 (3,318,366, 15,605,520)	12,956,038 (6,192,375, 27,341,593)	20,017,626 (9,585,679, 42,792,471)
ASPR (95% UI) per 100,000	586.41 (503.05, 678.16)	795.07 (687.50, 911.91)	706.22 (610.79, 812.27)
ASMR (95% UI) per 100,000	20.57 (5.12, 55.48)	28.81 (7.78, 72.97)	25.66 (6.84, 65.67)
ASDR (95% UI) per 100,000	372.44 (170.59, 822.78)	522.31 (250.04, 1100.88)	460.48 (218.29, 981.01)

DALYs, disability-adjusted life-years; ASPR, age-standardized prevalence rate; ASMR, age-standardized mortality rate; ASDR, age-standardized disability-adjusted life-year rate; 95% UI = 95% uncertainty intervals.

#### 3.1.2 Join-point regression analysis of ASR trend

**Figure 1:**
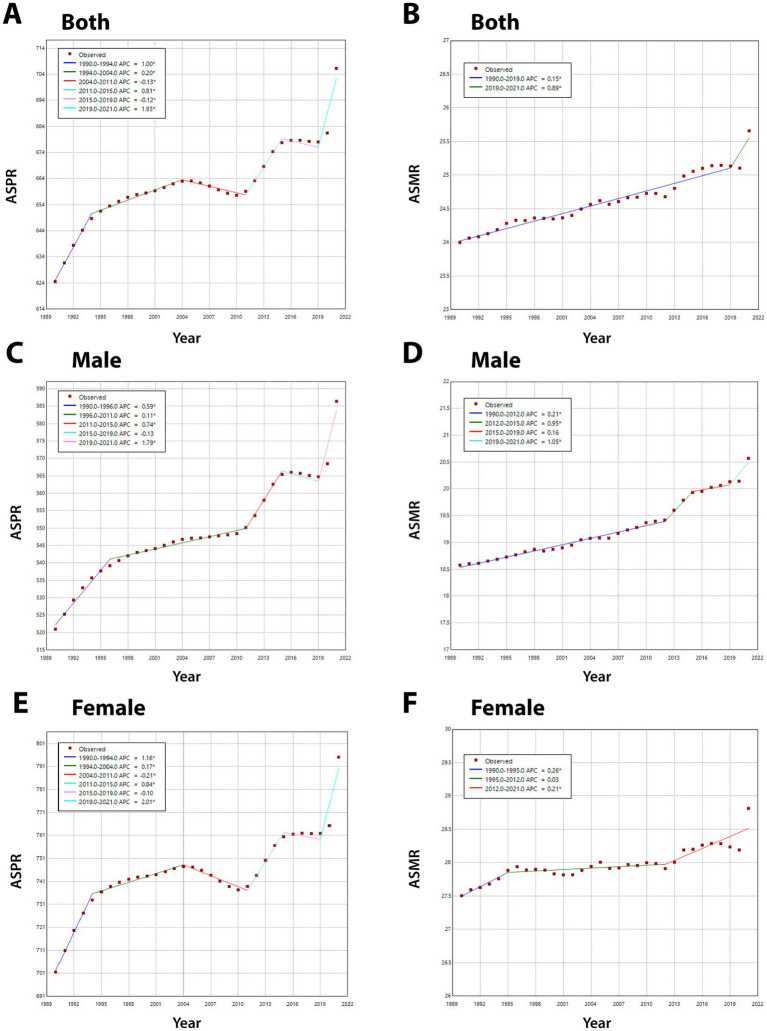
The join-point regression analysis of ASPR and ASMR in Alzheimer’s disease and other dementias. **(A,C,E)** Join-point regression analysis of ASPR in Alzheimer’s disease and other dementias in Asia for both genders **(A)**, male **(C)**, female **(E)** from 1990 to 2021. **(B,D,F)** Join-point regression analysis of ASMR in Alzheimer’s disease and other dementias in Asia for both genders **(B)**, male **(D)**, female **(F)** from 1990 to 2021. ASPR, age-standardized prevalence rate; ASMR, age-standardized mortality rate.

The segmental trends in the prevalence and mortality rates of AD and other dementias were assessed using join-point analysis (Figure 1). Notable shifts in the age-standardized prevalence rate were identified in the years 1994, 2004, 2011, 2015, and 2019, while a significant change in the age-standardized mortality rate was observed in 2019. The average annual percent change (AAPC) of the age-standardized prevalence rate over the entire period was 0.38% (95% UI: 0.35–0.40%), reflecting an overall upward trend. The most pronounced increases occurred during the periods of 1990–1994 (APC_1990–1994_ = 1.00%) and 2019–2021 (APC_2019–2021_ = 1.92%), whereas a gradual decline was observed between 2004–2011 (APC_2004-2011_ = −0.13%) and 2015–2019 (APC_2015–2019_ = −0.12%). In contrast, the age-standardized mortality rate for AD and other dementias in Asia exhibited a consistently stable upward trend (APC_1990–2019_ = 0.15%) between 1990–2019 and a significant rise between 2019–2021 (APC_2019–2021_ = 0.89%). The AAPC of the age-standardized mortality rate over the entire period was 0.2% (95% UI: 0.15–0.25%). Notably, a more rapid increase in the age-standardized mortality rate was observed in the male population compared to the female population, with an AAPC of 0.33% (95% UI: 0.26–0.39%) for males and 0.12% (95% UI: 0.26–0.39%) for females. The most significant increases were recorded for males during the periods 2012–2015 (APC_2012–2015_ = 0.95%) and 2019–2021 (APC_2019–2021_ = 1.05%), whereas the slowest growth was observed in females during 1995–2012 (APC_1995–2012_ = 0.03%).

### 3.2 Population distribution of AD and other dementias in Asia

**Figure 2:**
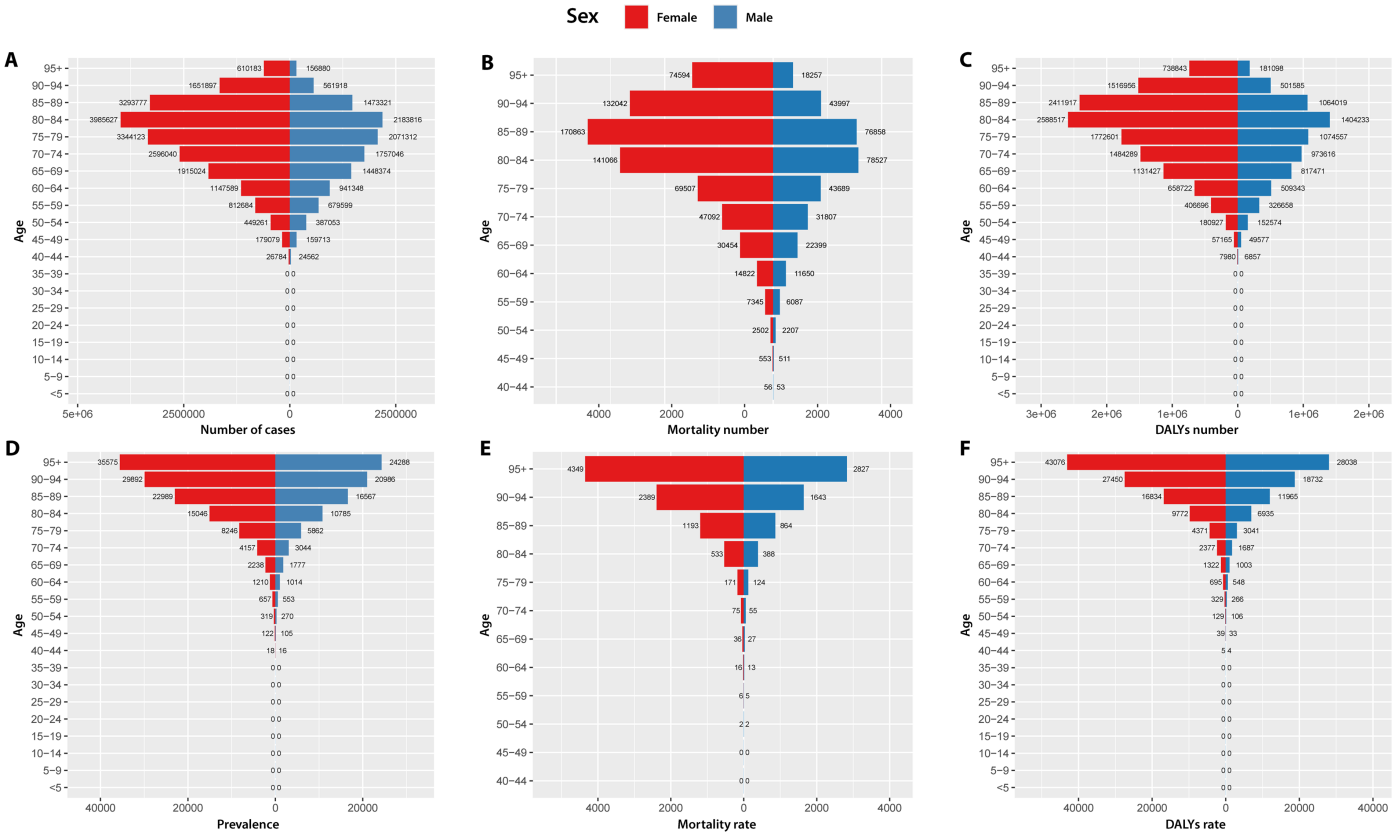
Number of cases **(A)**, mortality number **(B)**, DALYs number **(C)**, prevalence **(D)**, mortality rate **(E)**, and DALYs rate **(F)** due to Alzheimer’s disease and other dementias in Asia by gender in 2021. DALYs, disability-adjusted life years.

From a demographic perspective, in 2021, the overall burden of AD and other dementias was predominantly concentrated in the older adult age groups (>65 years old). As depicted in Figure 2, both the number of cases and the number of DALYs for AD and other dementias peak in the 80–84 age group for both sexes. The mortality number exhibits its zenith within the 85–89 age group for females and the 80–84 age group for males. Prevalence, mortality rate, and DALYs rate all demonstrate an increasing trend with advancing age, reaching their maximum values in the age group above 95 years for both male and female populations. In 2021, the number of cases, mortality number, DALYs number, prevalence rate, mortality rate, and DALY rate of AD and other dementias for females in Asia were all significantly higher than those for males.

### 3.3 Regional distribution of AD and other dementias in Asia

**Figure 3:**
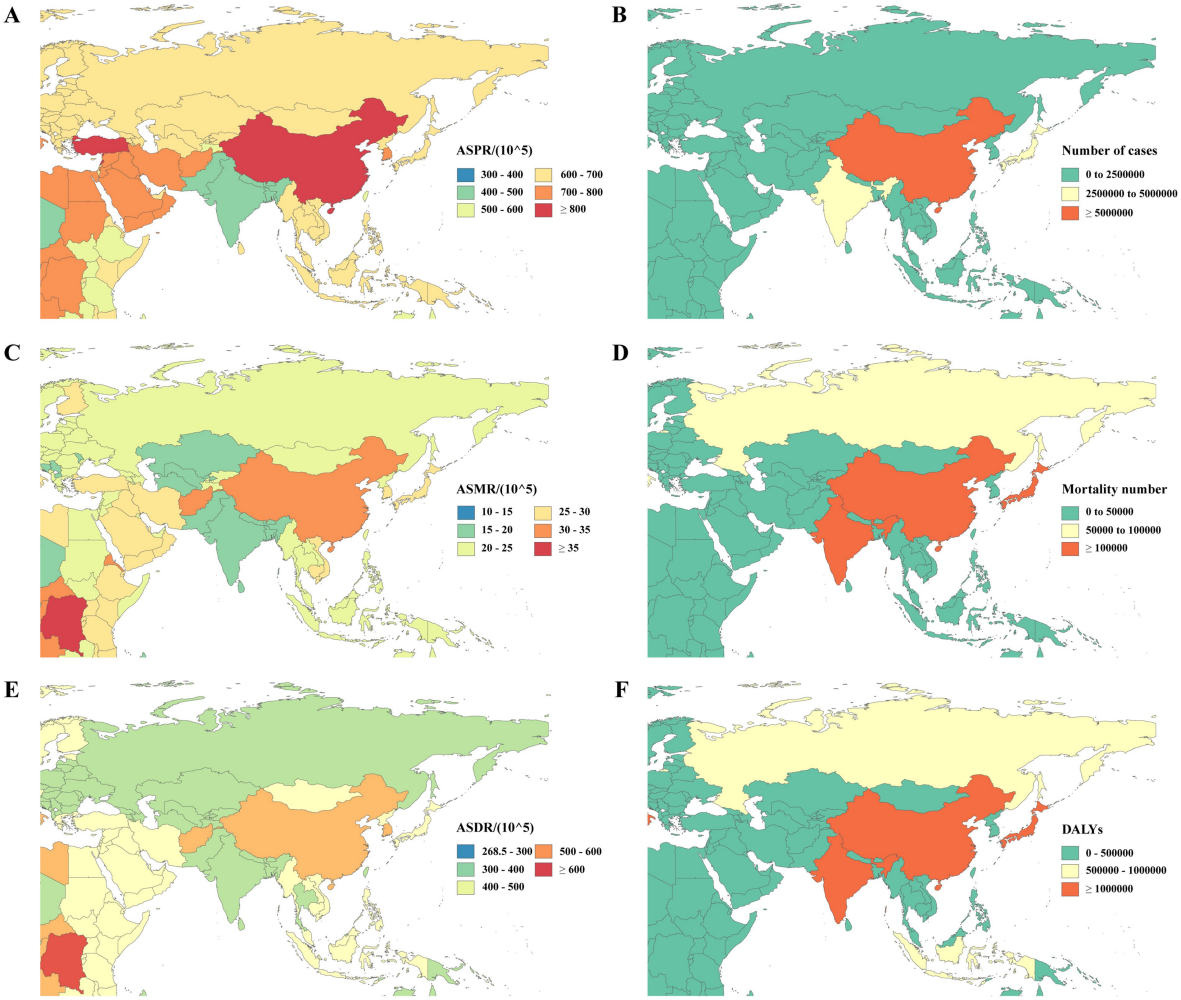
ASPR **(A)**, number of cases **(B)**, ASMR **(C)**, mortality number **(D)**, ASDR **(E)**, and DALYs **(F)** due to Alzheimer’s disease and other dementias in different countries and regions Asia in 2021. DALY, disability-adjusted life-year; ASPR, age-standardized prevalence rate; ASMR, age-standardized mortality rate; DALYs, disability-adjusted life years; ASDR, age-standardized DALY rate.

At the regional level, in 2021, the highest age-standardized prevalence rate (887.95 per 100,000 population [95% UI: 759.95–1027.48]), age-standardized mortality rate (30.41 per 100,000 [95% UI: 7.81–81.29]), age-standardized DALY rate (555.11 per 100,000 [95% UI: 267.58–1222.86]), number of cases (17414173.15 [95% UI 14854023.47–20142130.51]), mortality number (507658.77 [95% UI: 129183.83–1368539.61]), and DALYs (10359133.46 [95% UI: 580415.88–22833682.56]) were observed in the East Asia. The lowest age-standardized prevalence rate (437.07 per 100,000 [95% UI: 377.00–500.95]), age-standardized mortality rate (17.20 per 100,000 [95% UI: 4.10–47.28]), and age-standardized DALY rate (308.27 per 100,000 [95% UI: 135.52–684.96]) were observed in the South Asia. The minimum number of cases (398791.49 [95% UI: 345208.76–451918.05]), mortality number (11017.46 [95% UI: 2734.11–30437.33]), and DALYs (232274.49 [95% UI: 112588.80–498318.56]) were noted in Central Asia (Figure 3).

At the national level, in 2021, China reported the highest values for the number of cases (16990827.32 [95% UI: 14488494.04–19672741.19]), mortality number (491773.96 [95% UI: 124968.03–1330181.92]), DALYs (10072477.50 [95% UI: 4947154.11–22219153.71]), age-standardized prevalence rate (900.82 per 100,000 population [95% UI: 770.92–1,043.22]), age-standardized mortality rate (30.82 per 100,000 [95% UI: 7.88–82.43]), and age-standardized DALY rate (562.39 per 100,000 [95% UI: 271.16–1,238.81]), highlighting that the burden of AD and other dementias in China is the most severe in Asia. Conversely, Seychelles had the lowest values for the number of cases (635.89 [95% UI: 543.45–732.34]), mortality (17.95 [95% UI: 4.28–49.14]), and DALYs (371.27 [95% UI: 177.28–795.84]). The lowest age-standardized prevalence rate was observed in Pakistan (433.58 per 100,000 [95% UI: 374.32–499.28]), while India reported the lowest age-standardized mortality rate (16.98 per 100,000 [95% UI: 4.05–46.35]) and age-standardized DALY rate (305.73 per 100,000 [95% UI: 135.42–676.67]).

**Figure 4:**
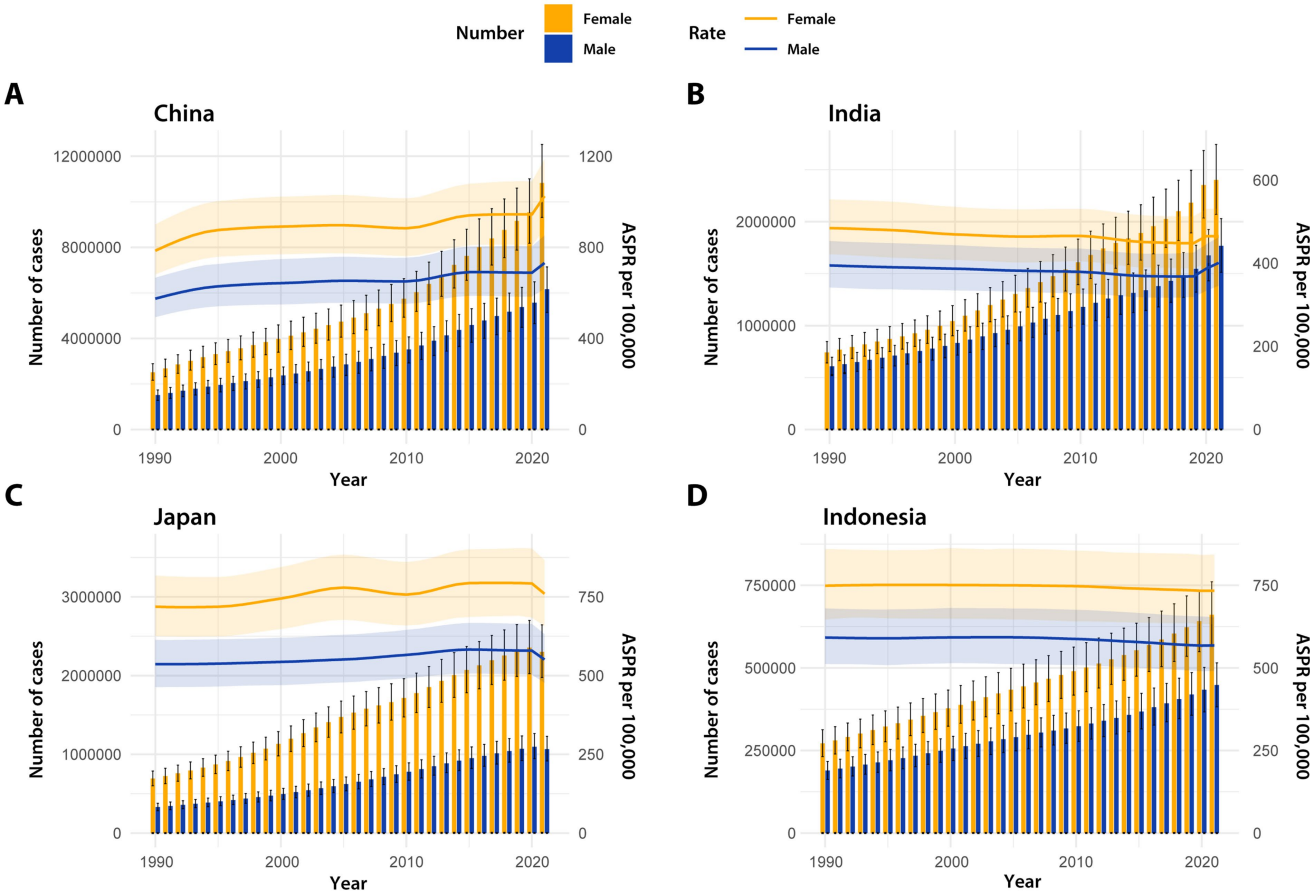
The number of cases and ASPR of Alzheimer’s disease and other dementias by year in China **(A)**, India **(B)**, Japan **(C)**, and Indonesia **(D)** from 1990 to 2021. ASPR, age-standardized prevalence rate.

Based on the aforementioned analysis, as shown in Table 2, we selected the four countries with the highest number of cases of AD and other dementias for further analysis, which are China, India (4169684.39 [95% UI: 3591611.95–4772663.15]), Japan (3367357.70 [95% UI: 2893950.69–3872083.71]), and Indonesia (1109363.84 [95% UI: 953356.39–1273165.62]). Figure 4 illustrates the trends of the number of cases and age-standardized prevalence rates among different genders in these four countries over the years. The results indicate that, consistent with studies in Asia, all four countries exhibit significant gender differences, with the burden of AD and other dementias being notably higher in female populations compared to their male counterparts. Regarding the number of cases, China, India, and Indonesia all show an increase over the years. However, Japan presented a divergent trend, with a continuous rise until peaking in 2020, only to experience a subsequent decline in 2021. Delving into the age-standardized prevalence rates, the data from each nation presents a varying result. China stands out with the highest age-standardized prevalence rate, exhibiting a predominantly ascending trajectory with the only exception being a modest downturn observed around the year 2010. India’s age-standardized prevalence rate has traced a gradual decline from 1990 to 2019, followed by a pronounced upswing in the years 2020–2021. In Japan, the age-standardized prevalence rate for females exhibits greater variability compared to that of males, often reflecting a distinct rhythm in comparison to the other three nations. The age-standardized prevalence rate for Japanese females predominantly displays an oscillating yet upward trend, in contrast to the more stable progression observed in males, which ascended gradually from 1990, peaked in 2015, and then began to recede. Indonesia’s age-standardized prevalence rate for both genders exhibited a period of relative equilibrium during the initial years of the study, only to undergo a more pronounced decline around 2006, albeit with varying degrees of steepness. Intriguingly, taking the number of cases in 2021 as an example, the ratio of female to male values from high to low is Japan (2.16), China (1.76), Indonesia (1.47), and India (1.36), which may suggest varying degrees of gender differences in disease burden across different countries.

**Table 2:** Prevalence of Alzheimer’s disease and other dementias by country and region in Asia, 2021.

Location		Prevalence (95% UI)	ASPR (95% UI)
Asia		31,857,009 (27,609,350, 36,564,142)	706.22 (610.79, 812.27)
Central Asia	Armenia	28,418 (24,402, 32,441)	651.84 (563.19, 741.68)
	Azerbaijan	47,757 (41,315, 54,594)	629.51 (541.15, 721.59)
	Georgia	43,615 (37,828, 49,553)	651.46 (562.89, 743.97)
	Kazakhstan	87,908 (75,726, 100,517)	634.04 (542.82, 724.42)
	Kyrgyzstan	23,637 (20,391, 26,813)	649.99 (562.04, 741.22)
	Mongolia	10,277 (8,905, 11,613)	660.02 (573.53, 749.23)
	Tajikistan	23,729 (20,338, 27,182)	597.35 (515.42, 684.84)
	Turkmenistan	18,834 (16,188, 21,360)	609.86 (523.2, 698.24)
	Republic of Uzbekistan	114,617 (98,143, 130,047)	605.59 (519.15, 691.73)
	Total	398,791 (345,209, 451,918)	626.81 (541.85, 713.52)
High-income Asia Pacific	Brunei Darussalam	1,331 (1,141, 1,519)	578.96 (496.58, 663.75)
	Japan	3,367,358 (2,893,951, 3,872,084)	674.1 (584.78, 770.24)
	Republic of Korea	697,045 (609,566, 789,739)	739.81 (647.7, 836.31)
	Singapore	43,791 (39,004, 48,432)	534.49 (474.45, 589.82)
	Total	4,109,525 (3,547,342, 4,692,562)	684.82 (596.97, 780.11)
South Asia	Bangladesh	516,766 (446,675, 592,095)	444.05 (383.22, 506.23)
	Bhutan	2,325 (1,996, 2,666)	433.7 (372.38, 497.86)
	India	4,169,684 (3,591,612, 4,772,663)	436.13 (376.02, 501.15)
	Nepal	83,342 (71,555, 95,053)	454.19 (392.41, 519.26)
	Pakistan	375,360 (321,684, 430,003)	433.58 (374.32, 499.28)
	Total	5,147,478 (4,444,260, 5,886,075)	437.07 (377, 500.95)
East Asia	China	16,990,827 (14,488,494, 19,672,741)	900.82 (770.92, 1043.22)
	Democratic People’s Republic of Korea	177,068 (151,286, 204,071)	618.92 (529.05, 710.41)
	Taiwan (Province of China)	246,278 (209,401, 276,216)	555.31 (472.14, 624.76)
	Total	17,414,173 (14,854,023, 20,142,131)	887.95 (759.95, 1027.48)
Southeast Asia	Cambodia	59,413 (50,607, 68,052)	658.61 (568.01, 754.43)
	Indonesia	1,109,364 (953,356, 1,273,166)	662.13 (571.11, 761.15)
	Lao People’s Democratic Republic	21,888 (18,988, 24,981)	655.85 (567.01, 749.46)
	Malaysia	153,807 (130,889, 175,275)	657.55 (561.66, 750.29)
	Maldives	1,794 (1,545, 2,056)	667.3 (572.03, 764.23)
	Mauritius	11,136 (9,549, 12,616)	657.17 (567.03, 748.3)
	Myanmar	257,641 (222,039, 292,332)	663.53 (571.48, 757.76)
	Philippines	419,363 (364,503, 477,877)	662.94 (575.83, 762.08)
	Seychelles	636 (543, 732)	643.49 (544.72, 741.31)
	Sri Lanka	152,889 (129,859, 175,784)	634.13 (545.74, 727.67)
	Thailand	659,437 (571,371, 751,935)	603.22 (522.58, 689.6)
	Timor-Leste	4,508 (3,824, 5,177)	664.45 (572.75, 759.71)
	Viet Nam	531,198 (453,586, 601,711)	649.42 (556.89, 744.39)
	Total	3,387,799 (2,922,771, 3,857,741)	644.38 (560.58, 737.69)

ASPR, age-standardized prevalence rate.

### 3.4 Prediction of prevalence and mortality over the next 15 years

**Figure 5:**
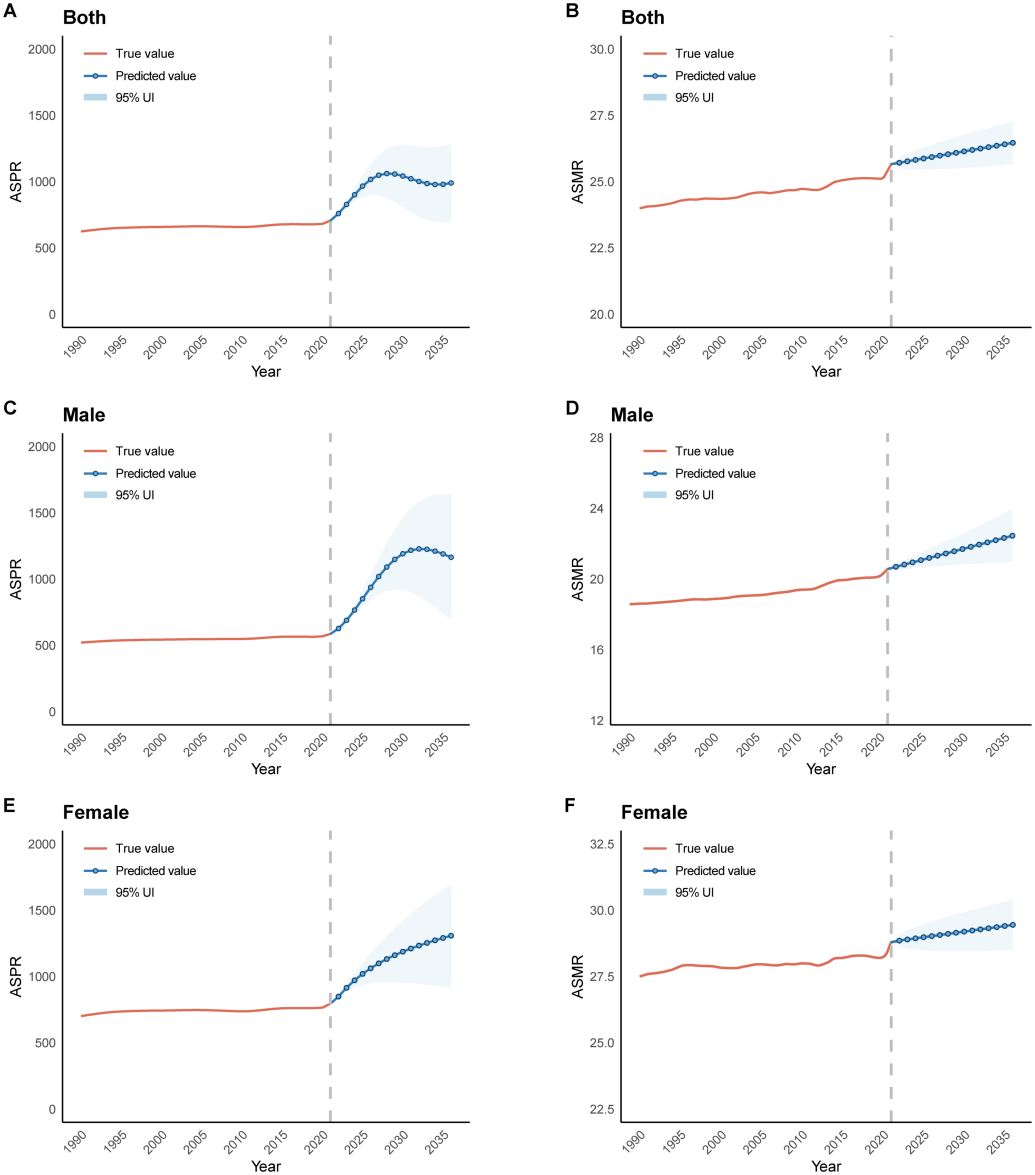
Predicted trends of prevalence **(A, C, E)** and mortality rate **(B, D, F)** in Asia in the next 15 years (2022–2036). ASPR, age-standardized prevalence rate; ASMR, age-standardized mortality rate.

The Autoregressive Integrated Moving Average (ARIMA) model was employed to quantitatively delineate the trends in age-standardized prevalence rate and age-standardized mortality rate for AD and other dementias in the Asian region over the next 15 years. The optimal model parameters, along with their corresponding AIC, BIC, and Ljung-Box test *p*-values, are detailed in Supplementary Table S1. The Ljung-Box test confirmed that all model p-values were greater than 0.05, indicating no significant autocorrelation in the residual series. This suggests that the models have adequately captured the information within the time series, demonstrating a good fit. As depicted in Figure 5, the analysis reveals that the age-standardized prevalence rate for AD and other dementias (per 100,000) in Asia is anticipated to exhibit a notable increase, rising from 759.16 in 2022 to 989.69 in 2036. Specifically, a swift upsurge in age-standardized prevalence rate is forecasted between 2022 and 2028, reaching a zenith of 1061.22 per 100,000 population in 2028, followed by a gradual decline. It is noteworthy that disparities exist among different age groups: the age-standardized prevalence rate for the male population in Asia is projected to mirror the trend of the overall population over the next 15 years generally, while the age-standardized prevalence rate for the female population is predicted to continue an upward trajectory. The age-standardized mortality rate for AD and other dementias (per 100,000) in the total Asian population is likewise anticipated to escalate over the next 15 years, increasing at a relatively stable pace from 25.71 in 2022 to 26.46 in 2036.

### 3.5 Risk factors of AD and other dementias

**Figure 6:**
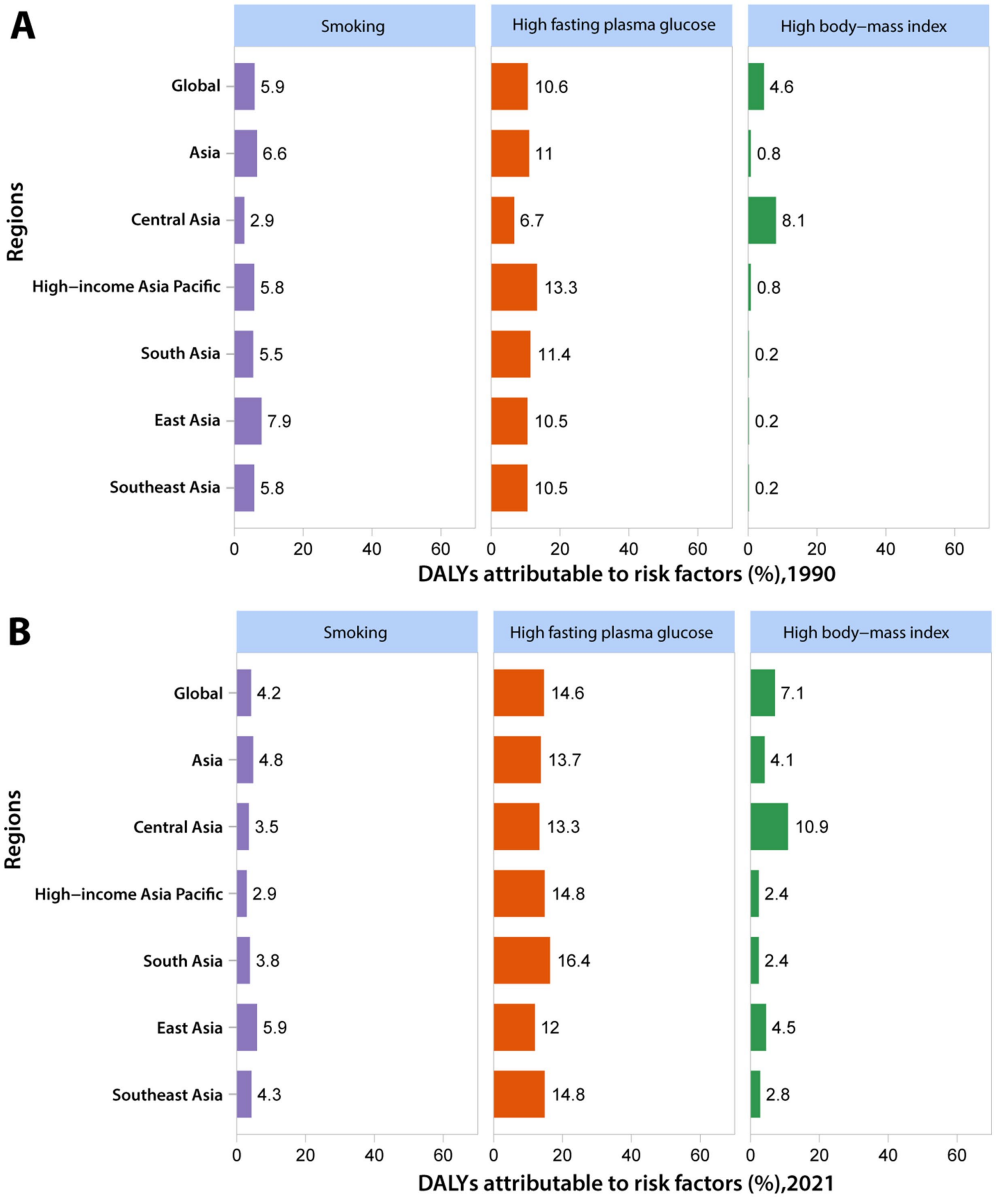
The attributable risk factors of Alzheimer’s disease and other dementias for both genders in Asia in 1990 **(A)** and 2021 **(B)**.

Three risk factors have been quantified for AD and other dementias, namely smoking, high FPG, and high BMI. In 2021, the attributable burden of DALYs for AD and other dementias across Asia was estimated at 21.0% (95% UI: 6.3–39.1%). The leading attributable risk factor was high FPG, accounting for 13.7% (95% UI: 1.1–27.4%) of the burden, followed by smoking (4.8% [95% UI: 3.4–6.2%]) and high BMI (4.1% [95% UI: 0.4–11.5%]). Compared to 1990, there has been a variable increase in the proportion of DALYs associated with high FPG and high BMI in Asia by 2021, whereas the proportion related to smoking has declined. At the regional level, East Asia had the highest proportion of DALYs attributable to smoking in 2021, South Asia had the largest proportion of DALYs attributable to high FPG, and Central Asia had the highest proportion of DALYs attributable to high BMI. It is noteworthy that in 2021, the proportion of DALYs attributable to smoking among Asian males (11.0% [95% UI: 7.8–14.1%]) was nearly eight times that of females (1.4% [95% UI: 7.8–14.1%]). Furthermore, the proportion of DALYs attributable to smoking in East Asian males (13.9% [95% UI: 10.0–17.8%]) ranks first among the regions studied. For the remaining two metabolic risk factors, in 2021, the proportion of DALYs attributable to high FPG was generally higher in males than in females across Asia and all regions, while the proportion attributable to high BMI was consistently lower in males than in females, aligning with global findings (Figure 6).

### 3.6 Association with the sociodemographic index

**Figure 7:**
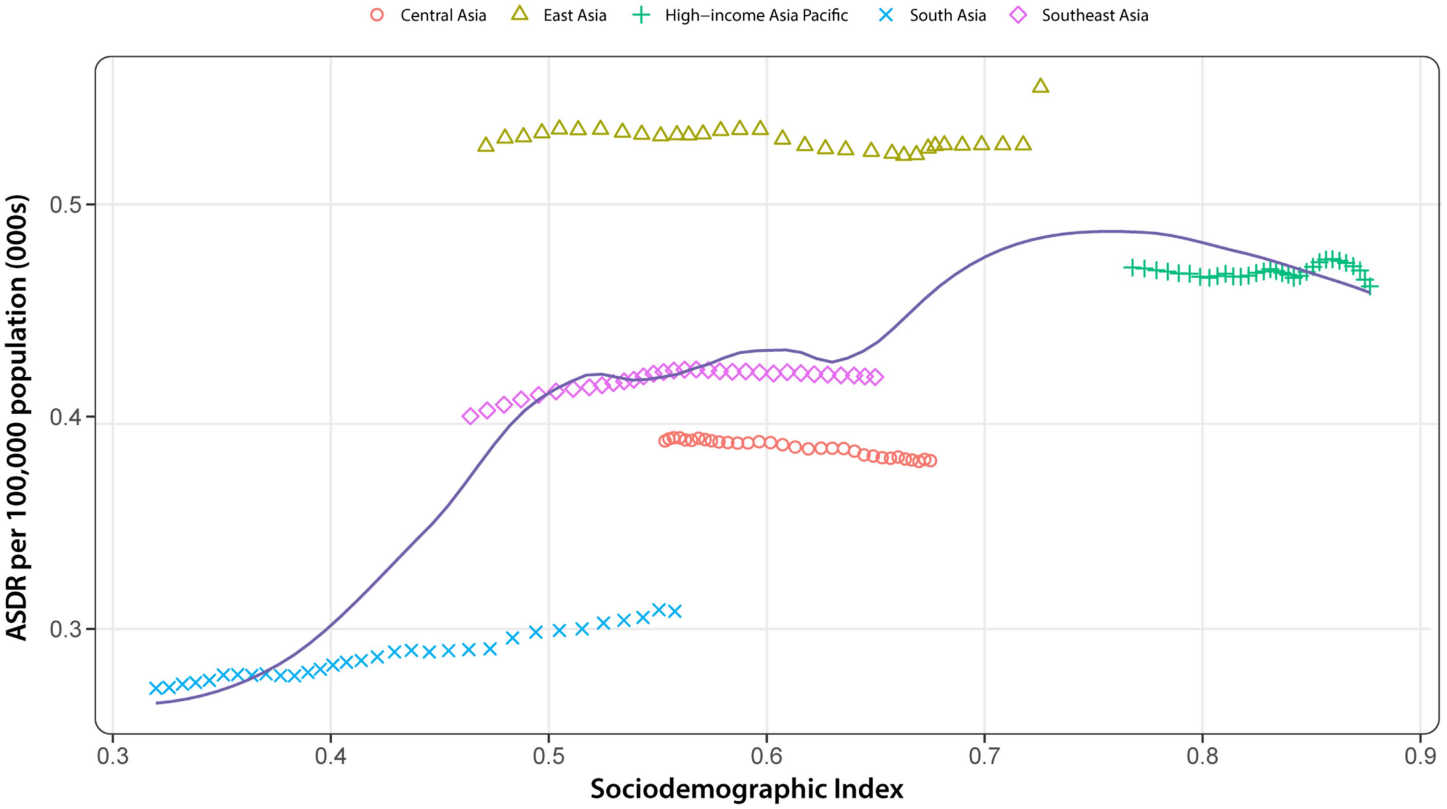
ASDR of Alzheimer’s disease and other dementias for 5 GBD regions in Asia by SDI, 1990–2021, with 32 data points plotted for each region. Expected values, based on the SDI and disease rates of all locations, are denoted by solid lines. Regions above the solid line indicate a burden exceeding expectations, while those below suggests a burden lower than expected. ASDR, age-standardized DALY rate; SDI, socio-demographic index.

As shown in Figure 7, our analysis from 1990 to 2021 revealed a complex nonlinear relationship between the SDI and age-standardized DALY rate for AD and other dementias across Asian societies. Initially, the age-standardized DALY rate exhibited exponential growth with an increase in SDI. However, once the SDI surpassed 0.5, the age-standardized DALY rate entered a phase of fluctuation within a defined range. This pattern continued until the SDI approached approximately 0.65, at which point the age-standardized DALY rate experienced a sharp increase, reaching its zenith around an SDI of 0.75, after which it began to gradually decline. From another perspective, there is significant heterogeneity in the age-standardized DALY rate caused by dementia at each SDI level. Notably, East Asia observed an age-standardized DALY rate that was higher than anticipated for its SDI from 1990 to 2021. In contrast, Central Asia experienced a burden that was lower than expected during the same period.

## 4 Discussion

### 4.1 Principal findings

In this study, based on data from the GBD 2021 study, we have provided up-to-date information of the prevalence, mortality, and DALY counts for AD and other dementias in Asia from 1990 to 2021, together with the age-standardized rates for 34 countries and territories. We found that AD and other dementias accounted for 31.86 million prevalent cases, 1.03 million deaths, and 20.02 million DALYs in Asia in 2021, representing astonishing increases of 250.44, 297.34, and 249.54% in comparison with that in 1990. This indicates a sharp rise in disease burden due to dementia. Consistently, the age-standardized prevalence rate, age-standardized mortality rate, and age-standardized DALY rate also exhibited varying degrees of growth, but the magnitude of increase is much smaller compared to the aforementioned indicators, which may be the result of population growth, aging, and increased life expectancy. Using past data, we have predicted the disease burden in Asia for the next 15 years. The results show that the future situation will be even more severe, especially with the continuous growth of age-standardized mortality rate. Overall, the above facts indicate that dementia has become an increasingly serious public health issue across Asia, underscoring the need for targeted public health interventions to address the escalating burden of these cognitive disorders.

### 4.2 Age differences

Global population aging has emerged as a major concern for medical and social demographics worldwide. Epidemiologic studies reveal that currently 11% of the world’s population is over the age of 60, and this figure is expected to double by 2050, accounting for 22% of the population (14). Furthermore, by 2050, the number of individuals aged 60 and above is predicted to surpass the youth population aged 10 to 24 (2.1 billion vs. 2 billion) (15). This trend is not limited to global statistics, it is also occurring in Asia, where rapid population growth and extended life expectancy have resulted in a significant aging population in this century. Additionally, declining fertility rates have further accelerated the aging process in certain countries.

Within the context of an aging society, the older adult population has seen a rapid increase in recent years. Aging is an inevitable and irreversible process associated with changes in the body’s appearance and functionality. Understanding the specific mechanisms of organismal aging poses a significant challenge, and current research has proposed 12 hallmarks of aging, encompassing genomic instability, telomere attrition, epigenetic alterations, loss of proteostasis, disabled macroautophagy, deregulated nutrient-sensing, mitochondrial dysfunction, cellular senescence, stem cell exhaustion, altered intercellular communication, chronic inflammation, and dysbiosis (16). Generally speaking, the incidence of age-related diseases increases with age, including primarily cancer, cardiovascular diseases, and neurodegenerative disorders. Aging itself is a significant risk factor for these health issues and plays a crucial role in their development. Furthermore, growing evidence has demonstrated that aging shares a common mechanistic foundation referred to as “hallmarks” with cancer (17), cardiovascular disease (CVD) (18), and neurodegenerative diseases (19), although the underlying relationships are highly complex. AD and other dementias, a category of neurodegenerative conditions experiencing the most rapid increase among age-related diseases (20), are projected to see a threefold increase in global prevalence by 2050. Additionally, in 2019, the annual societal cost of dementia worldwide was estimated at $1.313 trillion, equivalent to $23,796 per patient (21). In light of this evidence, dementia has become an urgent threat to global public health and well-being in an aging world without a doubt.

Therefore, as the global population continues to age, the burden of dementia will correspondingly increase. Previous studies on the burden of dementia have confirmed this grim reality (22). Asia, the most populous region in the world, is facing significant changes in its population structure, such as accelerated aging and slow growth, making it one of the regions most severely impacted by aging. In 2015, over half of the world’s older adult population resided in Asia, a proportion expected to rise to over 60% by 2050, exerting substantial pressure and burden on socio-economic systems such as healthcare, social security, and labor markets. In this context, the WHO must pay greater attention to the governance and prevention of dementia within the Asian region. This study utilizes the GBD research framework to conduct a comprehensive analysis of the disease burden of AD and other dementias among different genders, regions, and age groups in Asia from 1990 to 2021 and forecasts the trajectory of the disease burden over the next 15 years. Through this method, we aim to provide scientific evidence for the development of comprehensive prevention and intervention measures to alleviate the potential burden of dementia on individuals and society.

As the most significant risk factor affecting the occurrence and progression of AD and other dementias (3), the relationship between aging and disease burden has been mentioned earlier. In this study, there were virtually no cases of dementia among individuals under the age of 40 in Asia, and the disease burden primarily affects the older adult population above 65. Moreover, with increasing age, the prevalence, mortality rate, and DALYs rate due to AD and other dementias also rise gradually. Nevertheless, due to the shrinking population in the age group over 90 and the shortened life expectancy, the greatest number of cases (A), mortality number (B), and DALYs number observed were in the 80–90 age group.

### 4.3 Sex differences

By examining the burden of Alzheimer’s Disease and other dementias in Asia from 1990 to 2021, we observed that the number of cases, mortality number, DALYs, age-standardized prevalence rate, age-standardized mortality rate, and age-standardized DALY rate for females exceeded those for males in the Asian region, indicating a greater burden of dementia among Asian females compared to males. We believe that understanding the mechanisms behind the gender differences in AD and other dementias is a crucial step toward precision medicine, which will lead to more accurate diagnoses and more effective treatments for both men and women. From a sociological perspective, the longer life expectancy of women compared to men and their lower levels of education are non-negligible factors contributing to this phenomenon (23). However, an increasing amount of research indicates the importance of potential gender-specific pathological and physiological mechanisms. A recent review highlighted that the two main sources of biological sex differences are gonadal hormones and sex chromosomes, which interact with various factors during the aging process to create gender differences in disease onset and progression. Women experience menopause, during which estrogen levels fluctuate and may impact molecular pathways related to metabolism, inflammation, and other processes. Estrogen in women may reduce the levels of beta-amyloid (Aβ) by promoting the transport of amyloid precursor protein (APP) and may decrease the hyperphosphorylation of tau protein, but this effect weakens after menopause. Women have an extra X chromosome, and certain X chromosome genes are expressed at higher levels in females, which may play a protective or detrimental role in AD. Additionally, downregulation of the Y chromosome across brain regions may be associated with AD and other age-related diseases during the aging process (24). Gender-specific immune responses may affect the contribution of neuroinflammation to AD. For example, male microglia have higher MHC-1 gene expression, which may indicate a greater favorability for antigen presentation compared to females. Gender differences in metabolism may also impact brain health. Women may have early protection against metabolic dysfunction before menopause, but their susceptibility to cellular stress and diseases may increase after a decline in estrogen levels. Autophagy plays a critical role in clearing misfolded or aggregated proteins, and its dysregulation is associated with various age-related diseases, including neurodegenerative diseases. Females have lower baseline autophagy levels than males throughout their lives, which may result in more tau and amyloid accumulation. Research on the interaction between the gut microbiome and the brain is a relatively new field, and its composition varies between males and females, which may also affect the pathogenesis of neurodegenerative diseases (24). Given the complexity of the relationships, it is challenging to draw an accurate conclusion about gender differences. However, it is a known fact that by further elucidating the mechanisms of gender differences in the future, more effective treatment strategies can be implemented for patients with dementia.

### 4.4 Regions differences

In Asia, East Asia has the highest age-standardized prevalence rate, age-standardized mortality rate, and age-standardized DALY rate number of cases, mortality number, and DALYs with the number of dementia cases accounting for more than half of the total in Asia. The lowest age-standardized prevalence rate, age-standardized mortality rate, and age-standardized DALY rate were observed in South Asia, while the smallest number of cases, mortality numbers, and DALYs were noted in Central Asia. Additionally, among all neurological diseases, dementia ranks second in disease burden in East Asia and High-income Asia Pacific, second only to stroke. In contrast, this figure ranks fourth in Central Asia and Southeast Asia and fifth in South Asia (5). These differences are complex and influenced by a multitude of factors, including demographic structures, socioeconomics, healthcare, ethnic and cultural differences, and varying lifestyles.

In order to better understand the regional differences in disease burden, we selected four countries with the highest prevalence rates in five GBD regions for further analysis: China, India, Japan, and Indonesia. These countries all share the characteristic of having large populations, which is a common feature in many Asian countries and regions, resulting in high numbers of cases, mortality numbers, and DALYs. Furthermore, differences in population structure, levels of population aging, and the imbalance in economic development among these countries contribute to significant regional variations in the burden of dementia.

China now has the largest older adult population in the world, and by 2040, the number of people aged 60 and above in China is projected to increase to 402 million, accounting for approximately 28% of the population (25). As previously mentioned, age is the greatest risk factor for the onset of dementia, making China bear the largest disease burden in Asia. Additionally, under the sharp increase in China’s older adult population, the significant gap in the health and social care system may be contributing to the higher levels of the age-standardized prevalence rate. Corresponding measures must be implemented immediately to mitigate the impact of aging on China. These measures may include further strengthening primary care to improve access to healthcare services and promptly addressing the substantial gap in long-term care services. India is experiencing unprecedented demographic changes, with increased life expectancy and declining fertility rates significantly expanding the population aged 60 and above. With the continuous growth of India’s population, while that of China is already in decline, India’s degree of aging will gradually deepen and may potentially surpass China in the future. The level of social development is also an important factor contributing to regional differences in disease burden. Japan, as one of the most developed countries in Asia, had an SDI value of 0.87 in 2021 (high SDI region), while China, India, and Indonesia scored 0.72 (high-middle SDI region), 0.65 (middle SDI region), and 0.57 (low-middle SDI region), respectively. Japan is one of the countries with the most severe population aging, and the United Nations estimates that by 2050, people aged 65 and above will account for 42.0% of the population. A study has reported that Japan will experience the fastest-growing prevalence of AD from 2016 to 2026, which is largely consistent with our findings (26). However, thanks to its high economic level, Japan has a well-established healthcare and social security system, as well as a higher standard of care, which makes Japan’s age-standardized prevalence rate less severe than what might be expected given its aging population. As the fourth most populous country in the world, Indonesia currently has a relatively low degree of aging, with people aged 60 and above accounting for only 10% of the total population in 2019 (27).

In summary, China and Japan should take more action to address the economic burden and healthcare pressures caused by population aging, while India and Indonesia, being relatively early in their aging processes, should prepare adequately for achieving healthy aging in the future. These four countries come from four different GBD regions and have a certain level of regional representativeness. Therefore, our findings will help guide priority setting and resource allocation across different regions of Asia, particularly in countries with a high burden of AD and other dementias. Notably, due to the large population base and the uneven social development, low-and middle-income countries (LMICs) in Asia should be given high priority to mitigate the potential risks brought about by an aging society in the future. Specifically, these countries should actively improve policy formulation and resource allocation, establish sound community support networks and primary healthcare service systems, carry out health education to enhance citizens’ awareness and transform unhealthy lifestyle habits. In addition, they should proactively strengthen cooperation with high-income countries internationally, actively conduct local research related to dementia, and provide a basis for formulating targeted prevention and control strategies.

### 4.5 Risk factors

In the GBD 2021, only three risk factors were deemed to have sufficient evidence to establish a causal relationship with AD and other dementias: smoking, high FPG, and high BMI. Encouragingly, over the past three decades, there has been a 27.5% reduction in the number of smokers globally (28). Consistently, the proportion of DALYs attributable to smoking has significantly decreased during the study period. Strong evidence demonstrates that smokers have a higher risk of dementia compared to non-smokers, and those who quit smoking have a significantly lower risk of dementia than those who continue to smoke (29, 30). The link between smoking and cognitive impairment may be due to the connection between smoking and cardiovascular disease, and also because cigarette smoke contains neurotoxins, which also increase the risk. Furthermore, the elevation of AD risk attributable to smoking is more pronounced in apolipoprotein E ε4 noncarriers (31). In recent years, with the development of socioeconomics, changes in people’s living standards and lifestyles have made metabolic diseases a new challenge to human health, resulting in a significant upward trend in the disease burden attributable to high FPG and high BMI across various regions of Asia. Studies have confirmed that both diabetes patients and non-diabetes patients with elevated blood glucose levels are at increased risk of dementia (32). A systematic review of observational studies found that high glucose concentrations in the blood may exacerbate dementia-related neuropathology, such as greater amyloid-beta plaque burden, brain atrophy, and reduced cortical thickness (33). High blood glucose can stimulate the production of reactive oxygen species through plasma albumin, induce endothelial dysfunction, promote systemic inflammation, increase the number of activated microglia, and trigger neuroinflammation, thereby affecting cognitive and memory functions (34). Obesity (BMI ≥ 30.0 kg/m^2^) is also an increasingly serious global health issue. Numerous studies have examined the correlation between obesity and dementia, with results indicating that a higher BMI is associated with an increased risk of dementia (35–37). On the one hand, obesity is associated with the expression of AD-related genes (38). On the other, obesity often coexists with other metabolic issues such as insulin resistance, high blood glucose, and type-2 diabetes, sharing pathways leading to neurodegeneration, including oxidative stress, mitochondrial dysfunction, inflammation, adipokine dysregulation, and vascular impairment (39, 40). Psychologically, the shame experienced by individuals with high BMI can lead to elevated cortisol levels, inflammation, and negative health consequences, which may also be potentially associated with dementia (41). Therefore, measures such as reducing smoking, maintaining a healthy weight, and engaging in regular physical activity can effectively delay and reduce the incidence of dementia.

Chinese men consume approximately 40% of the world’s cigarettes, leading to the highest proportion of DALYs attributable to smoking in East Asia. Although this number has decreased in recent decades, the burden of death and disease caused by tobacco is still significant. The regional differences in the other two metabolic factors may be attributed to various aspects, including dietary habits, lifestyle, genetics, socioeconomics, culture, environment, medical resources, and health awareness. The threat posed by high FPG in South Asia has grown rapidly in the past, with the prevalence of diabetes steadily rising in Bangladesh, resulting in a loss of 4.0 million years of life (YLL) and 9.2 million productivity-adjusted life years (PALYs) due to diabetes (42). Similarly, in India, estimates from 2019 indicate that 77 million people have diabetes, with this number projected to rise to 134 million by 2045 (43). A systematic review found that genetic factors play a crucial part in the aforementioned phenomenon. Compared to other ethnic groups, Asian Indian people, broadly including individuals originating from India, Pakistan, Bangladesh, Sri Lanka, Afghanistan, Nepal, Bhutan, and the Maldives, have a specific phenotype characterized by lower BMI but higher levels of abdominal fat and insulin resistance, making them more susceptible to type 2 diabetes (44). High BMI has long been a significant factor contributing to the disease burden in Central Asia, with the proportion of the burden attributable to high BMI observed to be nearly 5 times higher than the High-income Asia Pacific and South Asia regions in 2021. For instance, in Pakistan, the ninth most obese country in the world, unconscious, high-density dietary consumption, and low physical activity are the main causes of overweight and obesity, leading to numerous obesity-related health issues (45). This trend is expected to continue to increase with the progress of urbanization and modernization, especially among the younger generation (46).

Multiple studies have revealed evidence supporting the idea that there are 11 other modifiable risk factors apart from age, gender, genetics, and family history that contribute to the development of dementia. These risk factors include having less education, head injury, physical inactivity, excessive alcohol consumption, hypertension, hearing loss, depression, infrequent social contact, air pollution, vision loss, and high cholesterol, which were identified and substantiated in the latest report by the Lancet Commission on dementia (23). Currently, there are no effective methods to prevent Alzheimer’s disease, and there is no cure available. Compared to the inherent risk factors for dementia mentioned earlier, lifestyle management and interventions targeting the modifiable risk factors mentioned above are crucial in reducing the risk of developing the disease (3). Therefore, it is imperative for relevant authorities to implement early screening for risk factors, as well as early diagnosis and treatment for related diseases. Concurrently, there should be an active dissemination of knowledge regarding disease prevention to enhance public health awareness.

### 4.6 SDI

In recent years, increasing attention has been paid to the study of the dementia burden in LMICs, with the aim of raising awareness of the potential disparities in diagnosis, treatment, and prognosis of the disease among different populations. Higher SDI regions, with better healthcare infrastructure and higher public awareness, may experience different challenges compared to lower SDI regions, where resource limitations and lower diagnostic capacity can lead to an underestimation of disease burden. Therefore, a multifaceted and targeted strategy must be implemented to prevent the potentially rapid increase in the dementia burden in LMICs and to actively improve the prevalence of dementia, its outcomes, and its impact on individuals and society in these countries (47).

Interestingly, in our study, the age-standardized DALY rate of Alzheimer’s disease and other dementias does not exhibit a monotonic relationship with the SDI. In Asia, apart from high SDI regions, the overall age-standardized DALY rate of AD and other dementias generally exhibits varying degrees of increase with the rise in SDI. Notably, the highest age-standardized DALY rate is observed in middle-high SDI regions, while the greatest increase in age-standardized DALY rate is seen in middle-low SDI areas. Typically, an increase in SDI is associated with an increase in life expectancy and a decrease in birth rate, leading to an aging population. Consequently, there is a higher burden of dementia. However, the results indicate that it is difficult to explain the trends between the disease burden and SDI solely based on population size and age. The underlying reasons may also involve factors such as healthcare quality, education levels, and public health policy, which may play different roles across countries and populations. Additionally, it must be acknowledged that there may be other factors not yet fully understood. Therefore, accurately interpreting these results poses challenges. The decline in age-standardized DALY rate in high SDI areas may be due to higher levels of healthcare quality and medical treatment that can provide better early intervention and care for patients, thereby reducing the burden of dementia. Moreover, advancements in lifestyle standards coupled with the increasing accessibility of higher education have further contributed to raising public awareness of dementia and mitigating associated risk factors. In contrast, the lower disease burden in low SDI regions may mainly probably owing to the lack of diagnostic capacity and shorter life expectancy (48). It is clear that dementia imposes a significant burden across regions with different SDI levels. Future work should continue to explore the impact of SDI stratification to better tailor prevention and resource allocation strategies, ensuring that interventions are appropriately targeted to the specific needs of each region.

### 4.7 Strengths and limitations of this study

One strength of this study is that it provides the most up-to-date and comprehensive estimates of the burden associated with AD and other dementias in the Asian region from 1990 to 2021, and offers predictions for the changes over the next 15 years, which holds significant implications for health services and policy.

The limitations of the study should also be acknowledged, which inevitably include some of the common limitations inherent in GBD studies, more detailed reports of which can be found in previous research. Firstly, the data used in this study are derived from GBD 2021 estimates, whose accuracy depends on the quality of the underlying data. The socioeconomic development in Asia is extremely uneven, and the collection and availability of dementia data may vary by region. The evidence still disproportionately comes from high-income countries, which may lead to underestimations of the burden. Additionally, the underdiagnosis and underreporting of Alzheimer’s disease and other dementias in LMICs and rural regions of Asia also contribute to underestimations. The low availability of biomarkers and the inapplicability of diagnostic tools in LMICs pose significant challenges for clinicians in detecting and diagnosing dementia in a timely manner. For patients themselves, poor awareness of dementia acts as a barrier to timely diagnosis. At a higher level, inadequate infrastructure and scarce resources in healthcare systems further hinder early diagnosis, resulting in insufficient diagnostic capacity (47, 49).

Secondly, the quality of cause-of-death data and verbal autopsy data relies on accurately coded death certificates and the expertise of the physicians who complete them. At the same time, comorbidities at the time of death further complicate this process, which may affect the accuracy of vital registration and verbal autopsy data sources.

Another key limitation of our analysis is the lack of uniform case definition criteria, which exposes the results to the influence of diagnosis and other biases in the original studies. In addition, leveraging the latest diagnostic criteria and consensus, such as the Revised Criteria published by the National Institute on Aging and the Alzheimer’s Association (NIA-AA) in 2024 or the ICD-11can further enhance the quality and persuasiveness of the data (50).

Fourthly, due to the constraints of the GBD framework, AD and other dementias were grouped together in our analysis. In fact, AD and other dementias can have distinct etiologies, clinical manifestations, and treatments. While this approach was necessary given the data limitations, it is important to acknowledge that this grouping may mask the potential heterogeneity and unique patterns of health impact between different types. Future research with more detailed subtype-specific data would be valuable in providing a more nuanced understanding of the disease burden and informing more targeted interventions.

Additionally, as previously mentioned, it is evident that risk factors related to dementia in the GBD 2021 are limited and thereby many other important modifiable risk factors were not assessed due to this limitation. With the continuous advancement in research on Alzheimer’s disease and other dementias, an increasing number of risk factors have been identified and confirmed to influence the onset, progression, and outcomes of these conditions. We hope that in the future, more risk factors can be quantified to provide a more comprehensive analysis of the disease burden.

## 5 Conclusion

In the 21st century, population aging is an inevitable trend in global demographic shift. As one of the most prevalent diseases among the older adults, AD and other dementias have become a significant public health challenge worldwide and will continue to pose an even greater issue in the future. Particularly in developing countries experiencing rapid demographic changes, the imbalance between the surge in medical needs and the lack of high-quality healthcare services has resulted in a substantial disease burden. Women and the older adult population should be the primary focus of attention. In conclusion, the burden of AD and other dementias in Asia, as reported in this study, along with their risk factors, provides the most up-to-date evidence to guide ongoing awareness and advocacy efforts. The findings emphasize that policymakers must develop and implement targeted interventions and preventative strategies to reduce the disease burden at both individual and population levels, ultimately promoting healthy aging for the entire human society.

## Data Availability

The original contributions presented in the study are included in the article/Supplementary material, further inquiries can be directed to the corresponding authors.

## Footnotes

^1^ http://ghdx.healthdata.org/gbd-results-tool
