# Supplementary material for: The current status, trends, and challenges of Alzheimer’s disease and other dementias in Asia (1990–2036)
Source: Front Public Health. 2025 Jun 10;13:1583339. doi: 10.3389/fpubh.2025.1583339 (PMC12185285; doi:10.3389/fpubh.2025.1583339)
Supplement: Supplementary file 1 [file Supplementary_file_1.docx]

| **Table S1 Autoregressive integrated moving average (ARIMA) model parameters and their corresponding AIC and BIC for prediction of ASPR, ASMR (per 100,000) of Alzheimer's disease and other dementias for the next 15 years in Asia** | | | | | |
| --- | --- | --- | --- | --- | --- |
| Measures | Sex | Parameters (p, d, q) | AIC | BIC | Ljung-Box test p-value |
| ASPR | Male | ARIMA (2, 1, 2) | 129.41 | 138.01 | 0.2683 |
|  | Female | ARIMA (1, 1, 2) | 190.2 | 197.19 | 0.6641 |
|  | Both | ARIMA (2, 1, 1) | 172.98 | 180.15 | 0.7406 |
| ASMR | Male | ARIMA (0, 2, 1) | -59.25 | -56.45 | 0.6254 |
|  | Female | ARIMA (0, 1, 0) | -38.54 | -35.67 | 0.993 |
|  | Both | ARIMA (0, 1, 0) | -48.47 | -45.61 | 0.9825 |

| **Table S2 Mortality of Alzheimer's disease and other dementias by country in Asia, 2021** | | | | | | |
| --- | --- | --- | --- | --- | --- | --- |
| Location | | |  | Mortality number(95%UI) |  | ASMR (95%UI) per 100,000 |
| Asia | | |  | 1026938.69(267220.27, 2616593.37) |  | 25.66(6.84,65.67) |
| Central Asia |  | Armenia |  | 902.65(224.08, 2385.95) |  | 21.34(5.34,56.32) |
|  |  | Azerbaijan |  | 1286.42(320.80, 3599.57) |  | 20.54(5.17,57.09) |
|  |  | Georgia |  | 1567.55(414.62, 4010.17) |  | 21.11(5.51,55.26) |
|  |  | Kazakhstan |  | 2135.99(513.53, 6150.80) |  | 19.44(4.58,55.94) |
|  |  | Kyrgyzstan |  | 632.21(157.02, 1634.69) |  | 20.09(5.07,52.52) |
|  |  | Mongolia |  | 247.71(61.71, 684.46) |  | 21.66(5.32,59.88) |
|  |  | Tajikistan |  | 672.83(163.48, 1788.32) |  | 21.59(5.44,58.06) |
|  |  | Turkmenistan |  | 567.86(137.29, 1587.63) |  | 19.71(4.78,54.98) |
|  |  | Uzbekistan |  | 3004.24(734.96, 8543.76) |  | 19.32(4.83,54.55) |
|  |  | Total |  | 11017.46(2734.11, 30437.33) |  | 20.07(5.03,55.69) |
| High-income Asia Pacific |  | Japan |  | 172806.94(50405.51, 398753.58) |  | 26.33(7.39,62.16) |
|  |  | Brunei Darussalam |  | 33.66(8.02, 90.73) |  | 23.27(5.71,62.03) |
|  |  | Republic of Korea |  | 25858.59(6916.69, 62627.11) |  | 29.02(7.87,69.71) |
|  |  | Singapore |  | 1459.74(391.10, 3566.55) |  | 17.90(4.76,44.10) |
|  |  | Total |  | 200158.92(57938.28, 463168.10) |  | 26.59(7.46,62.96) |
| South Asia |  | Bangladesh |  | 16559.52(3874.32, 49232.23) |  | 17.27(4.04, 50.94) |
|  |  | Bhutan |  | 94.95(23.05, 267.03) |  | 19.63(4.83, 54.56) |
|  |  | India |  | 133287.43(31383.59, 367632.69) |  | 16.98(4.05, 46.35) |
|  |  | Nepal |  | 2494.65(572.32, 6976.59) |  | 17.32(4.03, 48.09) |
|  |  | Pakistan |  | 12938.50(3049.61, 35773.05) |  | 19.59(4.73,53.82) |
|  |  | Total |  | 165375.06(39364.48, 454118.12) |  | 17.20(4.10, 47.28) |
| East Asia |  | China |  | 491773.96(124968.03, 1330181.92) |  | 30.82(7.88, 82.43) |
|  |  | Democratic People's Republic of Korea |  | 5834.09(1343.35, 16382.95) |  | 24.21(5.69, 67.83) |
|  |  | Taiwan (Province of China) |  | 10050.72(2642.28, 25165.72) |  | 21.13(5.44, 53.08) |
|  |  | Total |  | 507658.77(129183.83, 1368539.61) |  | 30.41(7.81, 81.29) |
| Southeast Asia |  | Cambodia |  | 1690.64(396.10, 4887.51) |  | 25.75(6.12, 71.34) |
|  |  | Indonesia |  | 26277.32(6326.28, 72878.41) |  | 22.97(5.62, 63.06) |
|  |  | Lao People's Democratic Republic |  | 547.31(127.63, 1527.28) |  | 21.60(5.14, 60.21) |
|  |  | Malaysia |  | 4715.67.67(1150.96, 12887.28) |  | 24.39(6.02, 65.59) |
|  |  | Maldives |  | 47.24(11.64, 127.42) |  | 19.55(4.80, 52.57) |
|  |  | Mauritius |  | 322.98(79.47, 872.57) |  | 20.30(5.00, 55.30) |
|  |  | Myanmar |  | 6858.07(1567.04, 19631.74) |  | 21.50(5.04, 60.71) |
|  |  | Philippines |  | 11051.83(2643.39, 27701.09) |  | 21.49(5.23, 54.38) |
|  |  | Seychelles |  | 17.95(4.28, 49.14) |  | 20.76(4.98, 56.32) |
|  |  | Sri Lanka |  | 3829.59(873.61, 10180.73) |  | 18.92(4.40, 49.86) |
|  |  | Thailand |  | 23490.53(6109.73, 60962.45) |  | 21.11(5.47, 54.92) |
|  |  | Timor-Leste |  | 105.55(25.37, 306.78) |  | 20.93(5.00, 59.12) |
|  |  | Viet Nam |  | 19281.09(4682.02, 53763.83) |  | 27.29(6.67, 75.83) |
|  |  | Total |  | 98372.97(24214.86, 259330.06) |  | 22.64(5.56, 59.23) |
| ASMR: Age-standardized Mortality rate; UI: Uncertainty interval | | | | | | |

| **Table S3 The DALYs of Alzheimer's disease and other dementias by country in Asia, 2021** | | | | | | |
| --- | --- | --- | --- | --- | --- | --- |
| Location | | |  | DALYs number(95%UI) |  | ASDR (95%UI) per 100,000 |
| Asia | | |  | 20017626.31(9585678.52,42792471.30) |  | 460.48(218.29,981.00) |
| Central Asia |  | Armenia |  | 17294.66(8319.29,36586.68) |  | 398.52(189.72,838.85) |
|  |  | Azerbaijan |  | 27684.89(13237.21,59577.35) |  | 384.43(179.15,829.85) |
|  |  | Georgia |  | 27728.67(13458.65,57375.27) |  | 398.83(191.96,827.23) |
|  |  | Kazakhstan |  | 48376.52(23936.44,105361.55) |  | 371.56(177.75,812.03) |
|  |  | Kyrgyzstan |  | 13596.26(6546.88,27881.04) |  | 386.99(186.91,800.00) |
|  |  | Mongolia |  | 5695.11(2781.99,12455.94) |  | 402.27(192.91,881.94) |
|  |  | Tajikistan |  | 14541.99(6783.31,31219.25) |  | 391.81(182.51,858.86) |
|  |  | Turkmenistan |  | 11362.73(5510.50,25239.73) |  | 373.23(181.83,824.24) |
|  |  | Uzbekistan |  | 65993.66(32043.46,141973.94) |  | 367.07(176.44,801.55) |
|  |  | Total |  | 232274.49(112588.80,498318,56) |  | 379.25(181.20,819.07) |
| High-income Asia Pacific |  | Japan |  | 2539293.652(1209734.79,5046913.08) |  | 456.00(221.79,920.17) |
|  |  | Brunei Darussalam |  | 781.93(371.07,1673.51) |  | 396.91(186.35,850.70) |
|  |  | Republic of Korea |  | 461797.18(218815.68,928411.31) |  | 500.05(234.92,1004.38) |
|  |  | Singapore |  | 26859.11(13116.93,54775.04) |  | 327.69(160.06,667.67) |
|  |  | Total |  | 3028731.88(1439614.46,6015524.17) |  | 461.33(222.39,928.28) |
| South Asia |  | Bangladesh |  | 342319.33(154927.19,804239.03) |  | 310.82(138.97,735.74) |
|  |  | Bhutan |  | 1747.34(765.62,4007.12) |  | 337.02(145.62,777.24) |
|  |  | India |  | 2787339.06(1236932.42,6211030.69) |  | 305.73(135.42,676.67) |
|  |  | Nepal |  | 53237.19(24524.08,119226.72) |  | 310.85(140.50,699.68) |
|  |  | Pakistan |  | 265492.28(113131.30,614292.61) |  | 333.84(140.09,758.92) |
|  |  | Total |  | 2086182.24(973510.03,4362204.89) |  | 418.63(193.30,883.66) |
| East Asia |  | China |  | 10072477.5(4947154.11,22219153.71) |  | 562.39(271.16,1238.81) |
|  |  | Democratic People's Republic of Korea |  | 117029.28(54009.92,258179.91) |  | 431.35(197.14,950.17) |
|  |  | Taiwan (Province of China) |  | 169626.68(79102.21,360771.39) |  | 372.14(175.26,790.05) |
|  |  | Total |  | 10359133.46(5080415.88,22833682.56) |  | 555.11(267.58,1222.86) |
| Southeast Asia |  | Cambodia |  | 38454.54(17789.13,89726.53) |  | 463.50(209.71,1084.98) |
|  |  | Indonesia |  | 633904.74(302564.43,1387129.98) |  | 422.00(194.37,933.56) |
|  |  | Lao People's Democratic Republic |  | 12588.34(5967.19,27894.64) |  | 407.47(187.60,907.38) |
|  |  | Malaysia |  | 98750.51(45297.57,216710.53) |  | 445.34(203.45, 976.51) |
|  |  | Maldives |  | 1002.42(491.71,2089.83) |  | 384.86(186.59,802.36) |
|  |  | Mauritius |  | 6516.25(3148.85,13909.97) |  | 390.08(186.92,832.38) |
|  |  | Myanmar |  | 149830.34(70323.67,330238.53) |  | 407.46(187.74,911.87) |
|  |  | Philippines |  | 245427.99(116780.95,509604.88) |  | 410.29(191.00,846.37) |
|  |  | Seychelles |  | 371.27(117.28,795,84) |  | 390.08(186.92,832.38) |
|  |  | Sri Lanka |  | 84903.47(40775.87,173229.27) |  | 369.00(177.21,764.88) |
|  |  | Thailand |  | 436132.60(204623.90,922677.62) |  | 395.44(185.94,837.14) |
|  |  | Timor-Leste |  | 2499.76(1202.69,5517.63) |  | 399.65(185.73,906.77) |
|  |  | Viet Nam |  | 372890.24(167940.53,839083.73) |  | 476.41(214.33,1068.35) |
|  |  | Total |  | 2086182.24(973510.03,4362204.89) |  | 418.63(193.30,883.66) |
| ASDR: Age-standardized Disability adjusted of life years rate; UI: Uncertainty interval | | | | | | |
